# Real-Time Wearable Cervical Posture Monitoring in Dentistry: A Prospective Usability Trial with Dental Students

**DOI:** 10.1055/s-0045-1812110

**Published:** 2025-10-22

**Authors:** Abubaker Qutieshat, Ritaj Al-Busaidi, Samiya Al-Ghammari, Al-Wejdan Koofan, Tuqa Al-Lawati, Mohammad S. Alrashdan, Polyxeni Petropoulou

**Affiliations:** 1 Department of Restorative Dentistry, College of Dental Medicine, University of Sharjah, Sharjah, United Arab Emirates; 2 Department of Restorative Dentistry, Oman Dental College, Muscat, Oman; 3 Department of Oral and Craniofacial Health Sciences, College of Dental Medicine, University of Sharjah, Sharjah, United Arab Emirates

**Keywords:** ergonomics, intervertebral disc, musculoskeletal pain, posture, wearable technology

## Abstract

**Objective:**

Dentists routinely adopt forward-lean postures that can lead to chronic spinal musculoskeletal disorders. Wearable real-time feedback may prompt microadjustments that preserve spinal health. This article aims to develop a wearable device for real-time detection of forwardleaning posture in dentistry, to assess its usability and alert rate during standardized student tasks, and to estimate cervical-disc fatigue lifetime from the measured headflexion profile using a simplified S-N model.

**Materials and Methods:**

An assistive device was constructed around an Arduino Uno interfaced with a three-axis FC-51 tilt-switch module, calibrated to trigger at > 30 degrees of head flexion. A piezo buzzer emitted pulsatile alerts when tilt exceeded the threshold. Twenty-four dental students (12 fourth year, 12 fifth year) wore the device during 30-minute simulated operative sessions. A research assistant logged each alert in real time. Postsession questionnaires (5-point Likert scale) assessed comfort, intrusiveness, distraction, workflow impact, and posture awareness. Qualitative feedback on power, alert modalities, and design refinements was collected. A fatigue model based on an S-N curve framework used measured angles to estimate years to cervical-disc fatigue under typical clinical exposure.

**Results:**

Head flexion averaged 42.7 degrees (standard deviation 9.4). The device generated a mean of 7.9 alerts per session, with no significant difference between year levels. Likert ratings indicated high comfort, low intrusiveness and distraction, minimal workflow disruption, and enhanced posture awareness; 79% of participants expressed willingness to adopt the device. Common suggestions included rechargeable power, multimodal alerts, slimmer enclosures, and customizable thresholds. The S-N fatigue model predicted disc fatigue onset at approximately 20.6 years for pure flexion and 16.0 years when lateral tilt was also considered, aligning with clinical data from the literature. Simulated use of the device, limiting “bad posture” to 1 minute per day, extended the model's fatigue lifetime to over 900 years.

**Conclusion:**

The goggle-mounted tilt-sensor device effectively detected and interrupted excessive forward-lean postures, was well accepted by users, and provided insights for ergonomic design improvements. Coupled with an S-N fatigue model, this approach offers both a practical intervention and a quantitative framework for mitigating career-long spinal risk in dentistry. Future work should validate long-term musculoskeletal outcomes and explore integration into clinical training.

## Introduction

Dentists face numerous occupational hazards daily, significantly impacting their overall health. Maintaining a robust musculoskeletal system is particularly critical in dentistry, given the physically and mentally demanding nature of the profession. Dentists must regularly execute precise hand movements, handle vibrating instruments, maintain static postures, utilize advanced psychomotor skills, and perform repetitive and monotonous tasks over extended periods. 1

In dental practice, muscle strain and joint angles vary depending on the posture adopted. 2 Consequently, extensive evidence demonstrates that musculoskeletal disorders and associated pain frequently limit dentists' productivity and quality of work 3 4 or even lead to premature retirement due to occupational disabilities. 5 6 Muscular imbalances and associated musculoskeletal disorders primarily arise from poor occupational postures, 7 8 largely resulting from repetitive and sustained awkward positioning. 9 Dentists are particularly vulnerable to disorders affecting the back, neck, and head due to their frequent forward-leaning postures throughout a typical workday. 9

Work-related musculoskeletal disorders affect a majority of dentists, with neck pain prevalence reported at 58%, 1 one-third seeking medical attention, 10 and 1.1% of practitioners developing cervical-disc herniation over a 5-year period. 11 Maintaining normal upright posture involves external body alignment and internal spinal alignment, which are fundamental to reducing the risk of spinal dysfunction. Downward flexion and rotation of the head, together with forward bending and rotation of the torso, substantially increase pressure on the spinal discs compared with natural resting positions. 12 Thus, sustained awkward postures that require stabilization by multiple head and torso muscle groups contribute significantly to musculoskeletal disorders. Regular posture adjustments to alleviate muscular tension are crucial in preventing fatigue across muscle groups. 9 13 From a biomechanics standpoint, these postures can be formalized using a kinematic description of the head–torso system, which helps link observed positions to mechanical loading. The fundamental principle in physics and engineering that any object's movement can be decomposed into rotation, translation, and deformation is well established for describing the six degrees of freedom of individual spinal segments considered as rigid bodies. 14 In practical ergonomic terms, the chairside deviations that most increase cervical loading are rotations away from neutral, particularly flexion/extension and lateral tilt, so strategies that minimize departures from neutral alignment are warranted.

Further, dentists also encounter upper-extremity problems such as carpal tunnel syndrome, characterized by numbness and tingling in the median nerve distribution and typically worse at night and with repetitive activity. Ergonomic contributors include repetitiveness, forceful exertions, mechanical or contact stress, posture, temperature, and vibration; practical prevention emphasizes maintaining neutral positions and reducing direct pressure over the carpal tunnel, supported by early recognition and education about ergonomic risk factors. 15 At the same time, sustained cervical flexion and static operating postures contribute to neck pain beginning in dental school, which has driven calls to integrate targeted ergonomic interventions into curricula; after a decade-long program, participants reported lower overall chronic pain prevalence and reduced neck pain intensity compared with national comparators, with earlier adoption associated with the greatest benefit. 16

Spinal musculoskeletal pain is recognized as a multifactorial, degenerative condition often linked to spinal degeneration and abnormal biomechanics that cause mechanical distortions within the central nervous system. 17 Symptoms typically manifest once the degenerative process has advanced. 18 Proactive identification and management of risk factors are therefore essential. Optimizing spinal alignment to resist gravitational compression is a logical approach, and developing a predictive model for cervical disc fatigue represents a useful step toward an evidence-based framework for clinicians and educators.

Given the significant musculoskeletal system overload, real-time monitoring and postural correction within the workplace have become urgent priorities. Effective monitoring can reduce the harmful effects of postures that deviate from proper alignment with the body's natural line of gravity. Posture represents a complex integration of responses to external stimuli, and wearable technology has emerged as a promising approach due to its adaptability and real-world applicability. 19 Wearable devices, also described as wearable sensors in existing literature, consist of electronic equipment seamlessly integrated into clothing or accessories, comfortably fitting the human body. Such devices have the potential to significantly enhance working conditions by early detection and correction of tendencies toward awkward or harmful postures. 20

Currently, several technological solutions for monitoring and recognizing sitting postures are proposed. With advancements in flexible and intelligent wearable technologies, there has been an increasing interest in wearable sensing systems due to their demonstrated accuracy, reliability, and convenience. Prior work has implemented wearable sensors for real-time posture monitoring, demonstrating practical value. 19 21 Effective sitting posture monitoring systems must provide immediate feedback mechanisms that facilitate corrective posture adjustments and support users in improving their ergonomic behavior. 21 To complement real-time monitoring technologies, recommended preventive strategies include stretching exercises before starting work, incorporating breaks throughout the day, adopting ergonomically appropriate postures during procedures, and minimizing repetitive movements. 22

Building on these insights and technological advances, this study had three objectives. First, to develop a low-cost, goggle-mounted wearable that detects excessive cervical flexion in real time. Second, to assess its usability and alert rate in dental students during 30-minute simulated operative sessions, with potential for adoption by practicing clinicians. Third, to establish a quantitative framework using an S-N model to estimate cumulative cervical loading and time-to-fatigue under typical dental postures. Together, these contributions offer both a practical wearable solution and a predictive tool to inform the development of ergonomic monitoring technologies in dentistry.

## Materials and Methods

In this single-center, prospective observational usability trial, an assistive device was implemented using an Arduino Uno microcontroller (a small, low-cost programmable board that reads sensor inputs and controls outputs) interfaced with a three-axis FC-51 tilt-switch module. The module's comparator output was connected to digital pin 2 (configured as “ *input_pullup* ”) to indicate when head/torso tilt exceeded a preset threshold. That threshold was adjusted via the module's blue trim potentiometer to approximately 30 degrees from vertical. A piezo buzzer on digital pin 8, powered alongside the Arduino by a 9 V battery carried in the wearer's scrub pocket, provided real-time ergonomic alerts.

Upon power-up, the Arduino sketch ran a one-time calibration routine, emitting “Calibrating device…” over the serial port and pausing for 1 second, to clear any residual tilt state. During normal operation, the comparator output was sampled every 50 ms and debounced over a 50 ms window. Whenever the signal transitioned to HIGH (tilt exceeded), a 1 kHz tone played for 200 ms; if excessive tilt persisted, additional 200 ms beeps repeated at 1 second intervals. Once the output returned to LOW, the buzzer was silenced immediately. All timing was handled via nonblocking “ *millis()* ” calls to ensure responsiveness. The complete Arduino sketch for sensor calibration, threshold detection, and pulsatile alert generation is provided in Fig. 1 .

**Fig. 1:**
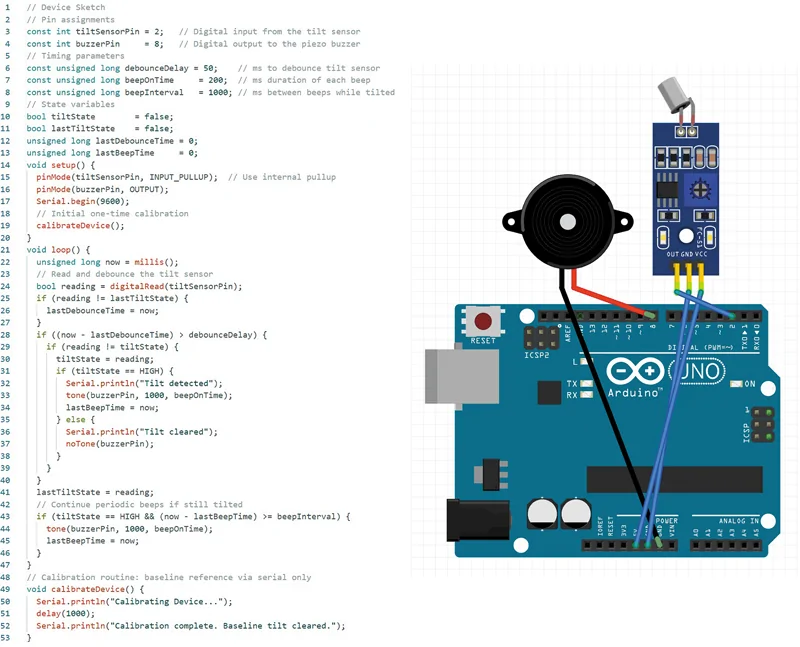
Fritzing schematic (v 1.0.1) of the device paired with the Arduino code for real-time posture monitoring and ergonomic feedback. The code implements sensor calibration, threshold-based tilt detection, and auditory alerts for posture deviations.

In Fig. 2 , the FC-51 module is shown affixed to a pair of protective goggles, with its three-wire cable routed neatly down the goggle arm. The module's two light-emitting diodes (LEDs) indicate posture state: when no LEDs are lit after power-up, the head is within the safe alignment zone; when the “tilt” LED illuminates (green), a forward-lean beyond 30 degrees has been detected.

**Fig. 2:**
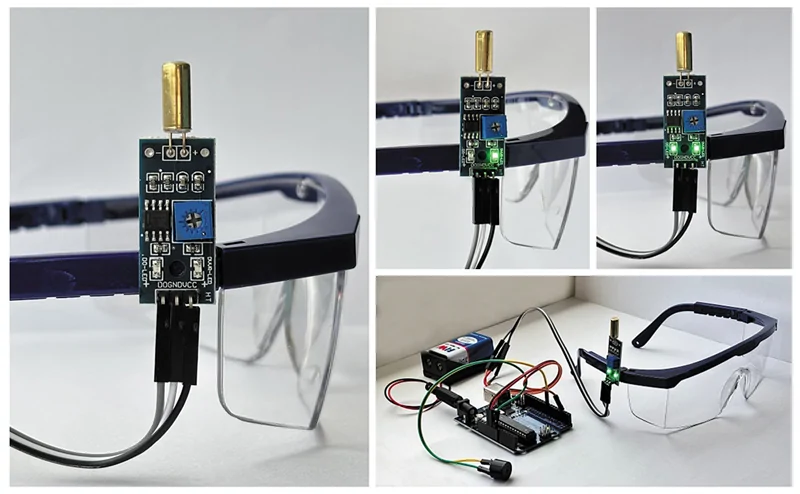
Mounting and actuation states of the FC-51 tilt sensor on protective goggles. The module is secured to the goggle frame (top panels) and connected via OUT, GND, and VCC lines to the microcontroller (bottom panel). In the normal posture (no tilt), only the power light-emitting diode (LED) is illuminated; on detecting excessive tilt, the comparator LED also lights, triggering the buzzer.

Twenty-four dental students (12 fourth year and 12 fifth-year) voluntarily participated in this study after responding to an email invitation and subsequently providing written informed consent. Ethical approval was granted by the institutional ethics committee prior to the start of the study. Consent was also obtained from all patients undergoing dental procedures performed by participants. Each participant wore the calibrated device continuously during a designated 30-minute segment of their clinical session, which included typical procedures such as drilling and restorative fillings ( Fig. 3 ).

**Fig. 3:**
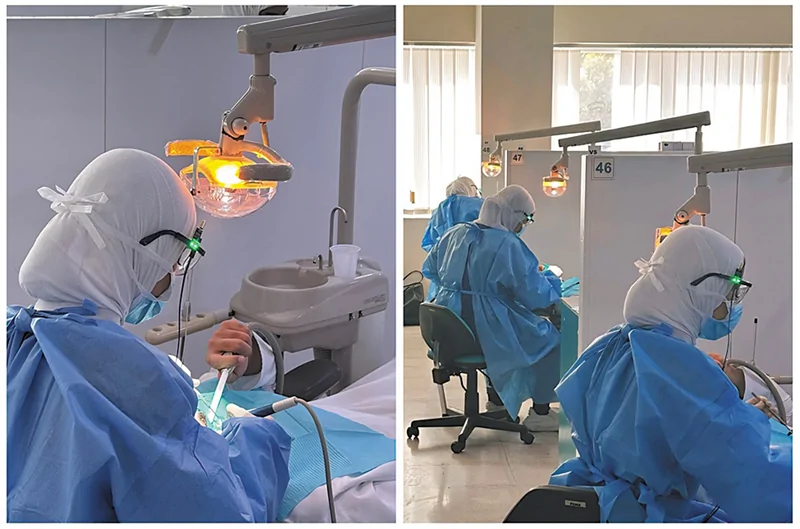
Dental students wearing the calibrated tilt-sensor device during live operative procedures. Left: a close-up view of a fourth-year student performing a restorative filling with the device affixed to protective goggles. Right: a wider shot showing multiple students in a clinic setting, each equipped with the posture-monitoring module.

Each 30-minute session was observed from a nonintrusive distance by a trained research assistant assigned to the operator. An alert was defined as any continuous period during which the buzzer was active, separated from adjacent events by a silent interval. Alert start and end times were logged using a smartphone lap timer with 1-second resolution, synchronized to the session clock at task start. For each session, two metrics were derived: alert count and total alert time (sum of all alert durations). Calibration beeps and any events outside the 30-minute window were excluded. Immediately after their sessions, participants completed a structured questionnaire assessing five dimensions on a 5-point Likert scale: comfort (1 = very uncomfortable, 5 = very comfortable), intrusiveness (1 = not intrusive, 5 = highly intrusive), distraction (1 = not distracting, 5 = highly distracting), workflow impact (1 = minimal impact, 5 = major impact), and baseline posture awareness (1 = low awareness, 5 = high awareness). Participants also indicated their likelihood of future adoption of the device (Yes, No, or Maybe). Data were analyzed using Python (version 3.9.10) and pandas to calculate means, standard deviations (SDs), and frequency distributions, both overall and separately by year group.

To develop a quantitative prediction model for cervical disc fatigue based on measured postural deviations, additional biomechanical variables were obtained from the literature, including average head mass (5 kg), gravitational acceleration (9.81 m/s ^2^ ), horizontal distance from the atlanto-occipital joint to the head's center of mass (0.06 m), and the moment arm length of cervical musculature (0.02 m). To contextualize the 30-degree alert threshold, we obtained sagittal head-flexion angles in a separate observational sample of 30 dental students during standardized operative tasks on manikins. In natural head position, the Frankfort horizontal (porion-orbitale) is approximately parallel to the ground; with standard spectacle alignment (horizontal temples and typical pantoscopic/face-form settings), the plane of protective glasses is likewise approximately parallel and can therefore serve as an external reference for head orientation. Angles were measured with the Apple Measure app (iOS 18.5) by aligning the phone along the temporal arm of the glasses and recording deviation from vertical. A single trained rater performed all measurements. This procedure was independent of the device trial described below. Using the measured angles (mean sagittal flexion, with additional lateral tilt of 15 degrees), muscle forces required to counteract head misalignment were calculated, enabling the estimation of cumulative mechanical stress and the predicted time to fatigue failure. These biomechanical calculations and assumptions are detailed further in the fatigue prediction model below.

### Cervical Disc Fatigue Prediction Model

Static equilibrium in the anteroposterior plane was used to characterize deviations from neutral alignment. Rotations about the *x* -axis (± Rx, flexion and extension) and the *z* -axis (± Rz, lateral tilt) were treated as nonneutral states that increase asymmetry of spinal tissue loading and muscular effort ( Fig. 4 ), reflecting movement away from the vertical neutral position.

**Fig. 4:**
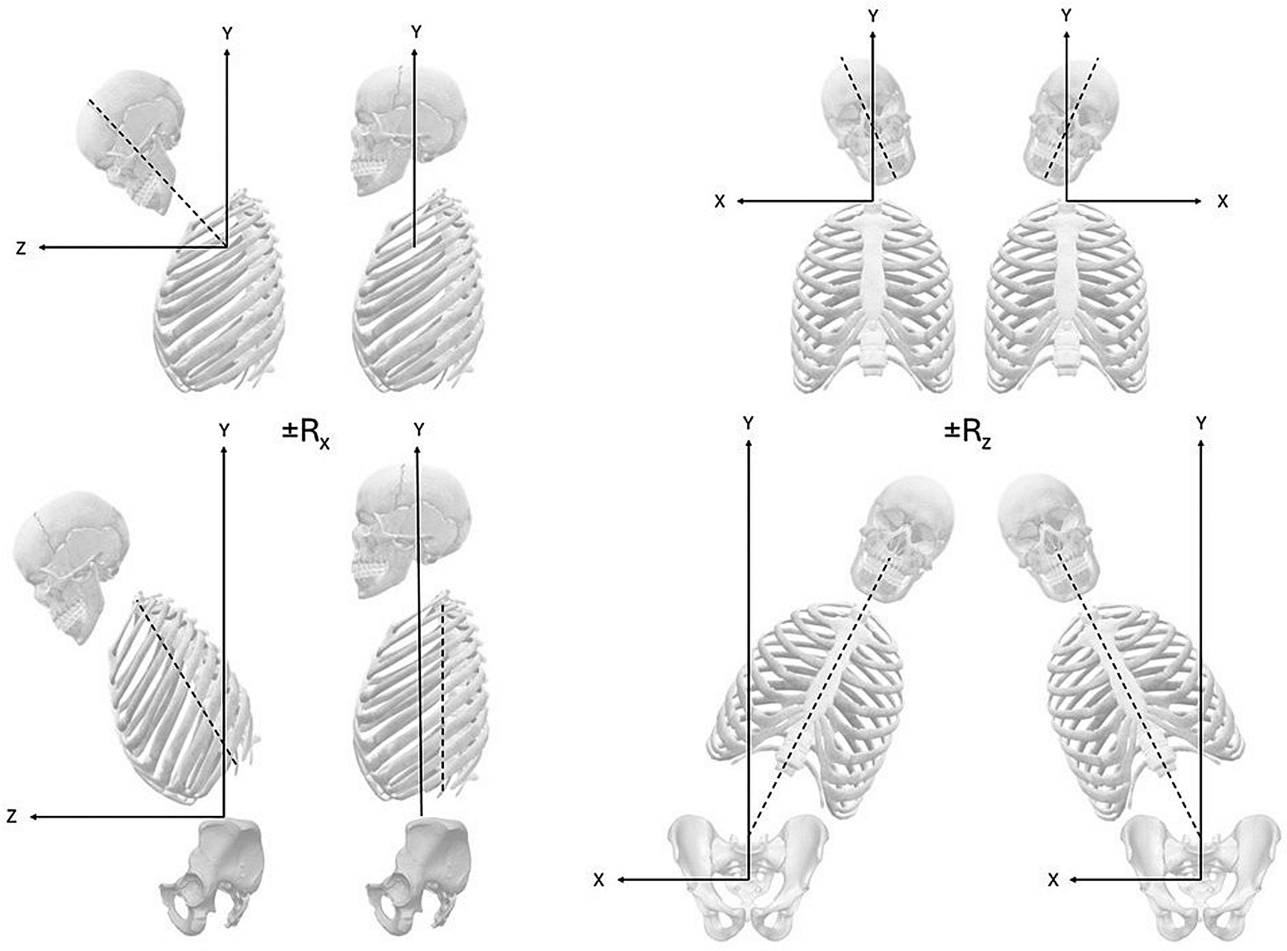
Rotational degrees of freedom of the head and torso. +Rx represents flexion (forward head lean and forward torso lean) about the lateral ( *x* ) axis. ± Rz represents lateral tilt of the head (ear moving toward shoulder) and torso (rib cage tilting left or right) about the anterior-posterior ( *z* ) axis.

For each segment, two axes of movement were considered. Each axis permitted two nonneutral directions, giving four single-axis deviations per segment; including combined deviations across both axes yielded eight unique nonneutral postures. Because the head and the torso can move independently, the combined head–torso posture space was modeled as a Cartesian product, yielding 64 unique postures.

To capture long-term risk of cervical disc fatigue under sustained forward head posture, an S-N fatigue framework was specified in which daily tissue damage scales nonlinearly with applied stress and subfatigue loading is repaired between workdays. Specifically, daily damage *d* was defined as



and the corresponding time to failure *T _fail_* in years follows:



Here, *F _muscle_* is the moment-derived muscle force required to resist sagittal head flexion and was calculated as



For combined sagittal flexion with lateral tilt, the resultant muscle force was



where mean head mass ( *m* ) = 5 kg, 23 gravitational acceleration ( *g* ) = 9.81 m/s ^2^ , horizontal distance from atlanto-occipital joint to head center of mass ( *d* ) = 0.06 m, and posterior cervical muscle moment arm ( *r* ) = 0.02 m. The sagittal flexion angle *θx* was parameterized by the baseline head-flexion measurement and is reported in the “Results” section; illustrative multiaxis scenarios used lateral tilt *θz* = 15 degrees. The fatigue limit for a healthy cervical disc was set to *F*_crit_ = 3,000 N, 24 25 and the S-N exponent for annulus fibrosus fatigue to *n* = 2.5. 26 27 Daily exposure *H* represented time above the 30-degree threshold in hours (1 hour/day of “bad” posture, 9 or 1/60 hour with device feedback), and *D*_year_ is the number of working days per year (240 days, allowing 1 month's vacation).

In static equilibrium, forward head flexion relative to the trunk increases the external flexion moment about the cervical spine; the posterior cervical muscles must generate greater extensor force to restore balance. With rising *θ _x_* , the modeled muscle force *F*_muscle_ increases, elevating joint reaction forces at cervical motion segments. Thus, time above a flexion threshold functions as a surrogate for cervical disc loading exposure in static and quasi-static tasks.

## Results

In the observational cohort ( *n* = 30), sagittal head-flexion averaged 42.7 degrees (SD 9.4). In the usability cohort ( *n* = 24; 12 Year 4, 12 Year 5), alerts per 30-minute session ranged from 2 to 18 (mean 7.88, SD 5.45); Year 4 averaged 8.00 (SD 5.58) and Year 5 averaged 7.75 (SD 5.56). Adoption intent was high: 19 of 24 respondents (79.2%) answered “Yes” and 5 of 24 (20.8%) answered “Maybe.” All 24 participants completed 5-point Likert ratings of usability and workflow impact: comfort 3.60 (SD 1.04), intrusiveness 2.36 (SD 1.04), distraction 2.20 (SD 1.04), workflow impact 1.64 (SD 0.76), and posture awareness 3.76 (SD 0.45).

Open-ended feedback prioritized power and alert modalities (rechargeable or coin-cell power; vibration and LED options; temporary mute), ergonomic refinements (routing cables within the goggle arm; a slimmer enclosure), and configurability (customizable angle thresholds; a battery-level indicator).

Using the baseline flexion angle from the observational cohort ( *θ _x_* = 42.7 degrees), the pure-flexion case yielded *F*_muscle_ ≈ 100 N, *d* ≈ 2.0 × 10 ^−4^ , and *T*_fail_ ≈ 20.6 years. When a lateral tilt was included ( *θ _z_* =15 degrees), the resultant force increased to *F*_total_ ≈ 110 N with *d* ≈ 2.6 × 10 ^−4^ , and *T*_fail_ ≈ 16.0 years. In a mitigation scenario limiting time above 30 degrees to 1 minute per day ( *H* = 1/60 hour; *D*_year_ = 240), the model yielded *d* ≈ 4.3 × 10 ^−6^ and *T*_fail_ ≈ 960 years. All estimates used *F*_crit_ = 3000 N and *n* = 2.5, and are reported with rounding consistent with input precision.

## Discussion

This study examined cervical posture in dental students in the context of the head–torso system's six degrees of freedom of individual spinal segments considered as rigid bodies, 14 with the chairside deviations most relevant to loading being rotations in flexion/extension (± Rx) and lateral tilt (± Rz). The observed mean sagittal head-flexion angle was 42.7 degrees (SD 9.4), consistent with reports from simulated sessions and with workload angles among practicing dentists. 16 28 The magnitude of this sustained flexion implies substantial time above common ergonomic targets such as 30 degrees, supporting the need for early, real-time feedback and training to reduce time spent in high-risk postures during clinical work. 28

The S-N analysis illustrates how realistic multiaxis posture deviations significantly accelerate fatigue onset, with the predicted timeframe closely matching epidemiological data among dentists. Fernandez de Grado et al found dentists with 15 to 25 years of practice had significantly increased odds of chronic spinal musculoskeletal pain (odds ratio [OR] = 2.1–2.4, *p* < 0.01), with further worsening after 25 years (OR = 3.9, *p* < 0.001; mean pain intensity > 5/10). 29 Similar findings were observed in Greek dentists, where chronic symptoms became prevalent after approximately 15 to 20 years of practice. 30 Additionally, a study among Belgian dentists indicated a significant decline in two-point discrimination ability in the dominant hand after 20 years of practice. 31 This consistency across multiple studies underscores the predictive model's clinical validity and emphasizes the importance of posture-focused ergonomic interventions.

In a mitigation scenario that limits time above 30 degrees to 1 minute per day, the model projects a dramatic extension of fatigue lifetime, effectively shifting the horizon from decades to centuries. This highlights how real-time postural monitoring and corrective feedback via wearable technology could help mitigate long-term spinal risk. Determining the accuracy and reliability of posture metrics as predictors remains essential before broader preventive applications are established. The model's practical utility should be evaluated in prospective clinical studies, and consistent definitions and measurements of head and torso posture remain a prerequisite. Given that some ergonomic guidance cites 20 degrees as a target for neck flexion, 32 the 30-degree device threshold used here should be viewed as a conservative, user-tolerant alert setting; exploring lower thresholds and task-specific targets may be warranted in future studies.

The empirical findings reinforce this interpretation. The mean sagittal head flexion observed in this cohort of dental students far exceeds the 20-degree ergonomic guideline for neck flexion, 32 and confirms that dental students routinely adopt forward-lean postures during operative tasks, underscoring the need for continuous ergonomic support. In female dentists, head inclination reached ≥ 39 degrees for half of clinical work periods and ≥ 49 degrees for 10% of the time, 28 while Katano et al documented flexion angles > 65 degrees during direct-view techniques. 33 Our device registered an average of nearly eight audible alerts per 30-minute session, confirming that these excessive postures are both frequent and sustained, conditions known to impair intervertebral-disc nutrition via loss of the disc “pump” mechanism, 34 and to accelerate degenerative changes under static loading. 35 Our findings also suggest that even experienced students frequently exceed safe tilt thresholds, and the absence of a substantial difference between Year 4 and Year 5 participants indicates that improper head posture persists throughout clinical training.

The S-N fatigue model developed here, which integrates measured tilt angles with disc fatigue thresholds, predicts structural fatigue onset at approximately 16 to 21 years of career-long exposure. This prediction aligns closely with epidemiological data showing significantly elevated odds of chronic spinal musculoskeletal pain after 15 to 25 years of practice and further rises beyond 25 years, 29 reinforcing posture as a reliable long-term predictor of spinal risk.

Traditional ergonomic interventions, such as saddle seats and magnification loupes, yield only modest reductions in neck flexion 36 ; if nonergonomic loupes are used, neck flexion can be even more pronounced, 37 and although soft cervical collars can limit extreme postures, they often cause discomfort. 38 In contrast, the wearable tilt-alert device tested here delivered real-time, point-of-deviation feedback that prompted microadjustments akin to “dynamic sitting” recommendations, 35 thereby restoring cyclical disc loading without restricting necessary clinical movement. High usability ratings (comfort = 3.60/5, intrusiveness = 2.36/5, workflow impact = 1.64/5) and strong adoption intent (79.2% “Yes”) indicate that unobtrusive, integrated feedback may overcome the compliance barriers seen with passive reminders. Participant suggestions such as rechargeable power, multimodal alerts, slimmer enclosures, and customizable thresholds echo calls for personalized, ergonomically optimized aids. 39 40 Iterative prototyping incorporating these features, alongside integration into dental curricula, 41 may amplify preventive impact. The qualitative feedback further highlights key areas for refinement, providing clear guidance for next-generation prototypes.

Several limitations warrant consideration. First, the trial involved a small convenience sample of 24 dental students in brief, simulated 30-minute sessions, which may not reflect the full range of clinical tasks or the experiences of fully trained practitioners. Second, usability ratings and alert counts were collected over a single exposure period, leaving open the possibility of novelty effects or Hawthorne bias influencing participant behavior and responses. Third, the FC-51 tilt module provides only a binary tilt signal at a fixed threshold and does not capture continuous angular variations or multisegment postures, which may underestimate the true extent of postural deviations. Fourth, the S-N fatigue model relies on assumed anthropometric parameters and disc fatigue limits drawn from the literature; it does not account for individual variations in spine geometry, tissue repair capacity, or cumulative exposure outside the clinical setting. Finally, no long-term clinical or musculoskeletal health outcomes were tracked.

Future work should pair multiaxis inertial sensing with automated on-device logging, examine threshold personalization and task-specific targets, and test whether posture-feedback training produces durable changes in clinical posture, reduced time above risky angles, and improved musculoskeletal outcomes. Prospective, longitudinal evaluation in authentic clinical settings will be essential to determine clinical benefit and to refine model parameters toward subject-specific estimates.

## Conclusion

A simple, goggle-mounted tilt-sensor device proved effective at detecting and interrupting excessive forward-lean postures, prompting timely corrective microadjustments during clinical tasks. Strong usability feedback and high willingness to adopt among dental trainees indicate its practicality for both education and practice. Integrating posture alerts with a fatigue-prediction framework aligns with observed patterns of spinal degeneration in dentistry, underscoring the value of real-time feedback in reducing cumulative mechanical stress. Future work should validate these results across broader clinician populations, add wireless data capture, and evaluate long-term musculoskeletal outcomes. Combining low-cost wearable alerts with predictive modeling offers a compelling strategy for enhancing ergonomic health in dental professionals.
